# An overview of colistin resistance, mobilized colistin resistance genes dissemination, global responses, and the alternatives to colistin: A review

**DOI:** 10.14202/vetworld.2019.1735-1746

**Published:** 2019-11-08

**Authors:** Mohammad H. Gharaibeh, Shoroq Q. Shatnawi

**Affiliations:** Department of Basic Veterinary Medical Science, Faculty of Veterinary Medicine, Jordan University of Science and Technology, P.O. Box 3030, Irbid 22110 Jordan

**Keywords:** animals, colistin alternatives, colistin resistance, Enterobacteriaceae, humans, mobilized colistin resistance-genes, one-health

## Abstract

Colistin, also known as polymyxin E, is an antimicrobial agent that is effective against a variety of Gram-negative bacilli, especially the Enterobacteriaceae family. Recently, the wide dissemination of colistin-resistance has brought strong attention to the scientific society because of its importance as the last resort for the treatment of carbapenem-resistant Enterobacteriaceae infections and its possible horizontal transmission. The mobilized colistin resistance (*mcr*) gene was identified as the gene responsible for unique colistin resistance. Indeed, despite many studies that have revealed a pan variation in the existence of this gene, not only for the *mcr* genes main group but also for its many subgroups, the problem is growing and worsening day after day. In this regard, this review paper is set to review the updated data that has been published up to the end of 2019 third quarter, especially when related to colistin resistance by the *mcr* genes. It will include the present status of colistin resistance worldwide, the *mcr* gene dissemination in different sectors, the discovery of the *mcr* variants, and the global plan to deal with the threat of antimicrobial resistance. In line with global awareness, and to stop antibiotic misuse and overuse, especially in agricultural animals, the study will further discuss in detail the latest alternatives to colistin use in animals, which may contribute to the elimination of inappropriate antibiotic use and to the help in preventing infections. This review will advance our understanding of colistin resistance, while supporting the efforts toward better stewardship, for the proper usage of antimicrobial drugs in humans, animals, and in the environment.

## Introduction

Colistin is an antimicrobial agent that belongs to the polymyxin antibiotic class, which is produced by a Gram-positive bacterium known as *Paenibacillus polymyxa*. This class consists of five polymyxins, A, B, C, D, and E, where polymyxin E (colistin) and polymyxin B are used clinically [1]. The colistin class of antibiotics is one of the last antibiotics that are used to treat Enterobacteriaceae infections in humans, such as colistin sulfate (CS) for oral and topical use, and colistin methanesulfonate (CMS) sodium for parenteral use [2]. In addition, colistin is a popular drug in the animal field, not only to treat infections caused by Enterobacteriaceae but it is also used as a growth promoter and a protective agent [3]. The rules for colistin usage are highly different from one place to another. For example, while the USA government has prohibited the clinical use of colistin in animal production and in human treatment, due to its nephrotoxicity within the human body [4], China is considered to be the world’s highest users of colistin in agriculture [5]. In addition, Germany, Portugal, Italy, and Estonia have shown a higher colistin use than in other European countries [6]. In the past few years, several review articles have highlighted the growing problem of colistin resistance worldwide, especially with *Escherichia coli* in the human community, but when regarding animals and other pathogens, the information is still scarce, due to the weak monitoring of its use. In this present review, the study’s goal was to provide the latest information related to colistin resistance by the mobilized colistin resistance (*mcr*) genes, in humans, animals, and in various pathogens. This review provides important insights into: (i) Demonstrating the present status of colistin resistance, the *mcr* gene emergency, and the global plan to deal with the threat of antimicrobial resistance (AMR); (ii) discussing various colistin researches, not only in the field of humans and farm animals but also in the aquaculture sector, while, at the same time, demonstrating the relationship between these sectors in the dissemination of the plasmid resistance gene *mcr*; (iii) illustrating the *mcr* gene dissemination by trade and travel and the discovery of the *mcr* variants starting from *mcr*-1 up to *mcr*-9; and (iv) presenting suggested alternatives to colistin for the treatment of infectious diseases.

## Colistin and its Resistance Emergency in Animals

The *mcr* genes are plasmid-borne genes that contribute to colistin resistance. To date, nine *mcr* variants have been described, as shown in Table-1, (*mcr*-1, -2, -3, -4, -5, -6, -7, -8, and -9). As mentioned earlier, many recent studies have shown the wide distribution of *mcr*-genes in animals. Rhouma *et al*. [3] indicated that the extensive use of colistin in production animals was recognized as the responsible agent for the emergence and dissemination of the plasmid-borne colistin resistance gene *mcr*-1. Recently, Liu *et al*. [7] showed evidence for the isolation of Gram-negative bacteria from resistant animals to colistin. Furthermore, many studies have detected the existence of the *mcr*-1 gene in different animal food species. For example, Meinersmann *et al*. [8] reported this gene in *E. coli* isolate of pig cecal contents from the USA. Also, Huang *et al*. [9] reported the *mcr*-1 gene from *E. coli* isolates of animal food origins (chickens and pigs) from China [9]. In addition, Barbieri *et al*. identified the *mcr*-1 gene in poultry samples from the year 2010. They examined 980 Avian pathogenic *E. coli* that was isolated from poultry and that was suffering from colibacillosis, and for the comparison analyses, they compared an additional 220 sets of non-infected avian fecal E. coli. The *mcr*-1 gene was reported in 12 isolates that were recovered from diseased production birds from China and Egypt. Remarkably, the date for the positive *mcr*-1 gene from the Egypt isolates was back in 2010. On the other hand, no *mcr* genes were reported in any of the healthy fecal isolates [10]. In other studies, the *mcr*-1 gene was identified in *E. coli* when it was isolated from diseased chickens and cows suffering from subclinical mastitis in Egypt [10-12]. Barbieri *et al*. [10] suggested that the huge usage of colistin in animal agriculture, and its ready application as a therapeutical agent for colibacillosis and other infections in rabbits and calves, was responsible for the emergence of *mcr*-1 in Egypt [10]. The full *mcr*-1 gene isolates were sequenced and compared with the sequences currently described in NCBI [https://www.ncbi.nlm.nih.gov/nuccore/ku886144] and in Arcilla *et al*. [13]. Furthermore, the phenotypes and the genotypes of the *mcr*-1 positive isolates were determined as being colistin resistance and as extended-spectrum beta-lactam (ESBL). Earlier, Quesada *et al*. [14] proved that Spain detected *mcr*-1 in *E. coli* and in *Salmonella enterica* when isolated from farm animals. Interestingly, Hernández *et al*. [15] reported on a coexisting of *mcr*-1 with *mcr*-3 on the same IncHI2 plasmid which was reported in an *E. coli* strain cured of cow feces in a slaughterhouse in Spain. Haenni *et al*. [16] identified the presence of a special IncHI2/ST4 plasmid that was colocalizing *mcr*-1 and ESBL genes in French veal calve isolates of *E. coli* strains. A research study in China investigated the colistin spread in farm animals and revealed that *mcr*-1 and *mcr*-2 were detected in cattle, pig, and chicken origins of *E. coli* isolate, where *mcr*-1 was the higher percentage [17]. Moreover, in the same study, a cooccurrence of *mcr*-1 and *mcr*-2 was reported, but with a low ratio when in comparison to the *mcr*-1 and *mcr*-2 percentages [17]. Alba *et al*. [18] reported on *mcr*-1 and *mcr*-2 in *E. coli* from turkey origins in Italy. The cooccurrence of *mcr* genes was reported in Spain [19], where the *mcr*-1, *mcr*-4, and *mcr*-5 genes were found in multidrug-resistant (MDR) ST10 enterotoxigenic and Shiga toxin-producing *E. coli* from swine with post-weaning diarrhea.

**Table-1:** The first identification of the *mcr* genes by time and area.

Gene	Year	Country	Bacteria	Sample origin	Length	References
MCR-1	2016	China	*E. coli*	Animal, Human, Food	1626 bp	[7]
MCR-2	2016	Belgium	*E. coli*	Animal	1617 bp	[28]
MCR-3	2017	China	*E. coli*	Animal	1626 bp	[24]
MCR-4	2017	Italy, Spain and Belgium	*E. coli*, S*almonella*	Animal	1626 bp	[25]
MCR-5	2017	Germany	*Salmonella*	Animal, Food	1644 bp	[26]
MCR-6	2017	UK	*M. pluranimalium*	Animal	1617 bp	[27]
MCR-7.1	2018	China	*K. pneumoniae*	Animal	1620 bp	[21]
MCR-8	2018	China	*K. pneumoniae*	Animal	ND	[22]
MCR-9	2019	USA	*S.* Typhimurium	Human	ND	[23]

*E. coli=Escherichia coli, M. pluranimalium=Moraxella pluranimalium*, *K. pneumoniae=Klebsiella pneumonia*, *Salmonella* Typhimurium*=Salmonella* Typhimurium

## *Mcr* Gene Mechanisms and their Members

In 2016, the first report to show the emergence of the plasmid-mediated polymyxin resistance mechanism, *mcr*-1, in Enterobacteriaceae, was discovered in China [7]. The next step was to determine the mechanisms of plasmid-mediated colistin resistance and the *mcr*-1 gene. This was reported by Hinchliffe *et al*. [20] when they proved that the *mcr*-1 gene works to change the target of colistin through the action of the enzyme, phosphoethanolamine transferase, which transfers glucosamine from lipid A, while the negative charge of lipid A is reduced; consequently, colistin cannot connect. Interestingly, *mcr*-1 was not the only member of the *mcr*-genes, because a series of recent studies have indicated the *mcr*-gene presence is in humans and animals, and they have revealed nine different *mcr* genes. Four of them (*mcr*-1, 3, 7.1, and 8) were first detected in China, and four genes (*mcr*-2, 4, 5, and 6) were reported in Europe. Finally, *mcr*-9 was reported in the USA, starting from 2016 up to 2019, as shown in Table-1 [7,21-28]. Of the recently detected genes, *mcr*-7.1 (1620bp) and *mcr*-8 have been reported in China in 2018, and both were hosted by *Klebsiella pneumoniae* from human and animal origins [21,22]. In addition, a recent study by Carroll *et al*. [23] identified *mcr*-9 in an MDR *S. enterica* serotype Typhimurium isolate, which was colistin-susceptible in the USA and demonstrated the phylogenic tree connects between *mcr*-1 and *mcr*-9.

## The Discovery of *mcr* Variants

Recently, some unknown selective pressure in the environmental section and in the animal field and human sectors was considered as being the responsible agents for the constant evolution of the *mcr* gene, which ended by producing the *mcr* variants, as suggested by Sun *et al*. [29]. However, within a short period of time, the *mcr* genes included several variants nearly all around the world, for instance, *mcr*-1 has 13 variants that are different by one amino acid from *mcr*-1 (*mcr*-1.1 to *mcr*-1.13) [30,31]. Recently, Wise *et al*. [32] collected 908 Enterobacteriaceae isolates worldwide, and the results revealed that while 22 isolates carried the basic *mcr*-1 gene, one of the isolates had the *mcr*-1 gene with a single amino acid variant *mcr*-1.1 and another isolate held *mcr*-1.5. The 13 *mcr*-1 subgroups have already been described in several countries, differing from *mcr*-1 by only one nucleotide. In Thailand, three isolates of *mcr*-3.1 and one isolate of *mcr*-3.2 were reported. Likewise, Ling *et al*. [33], in China conferred polymyxin resistance by the presence of *mcr*-3.2 in both *Aeromonas salmonicida* and *E. coli*. During the same year, *mcr*-3.7 was identified by Teo *et al*. [34], in Singapore, and it was not resistant to colistin or polymyxin B. This was while Chavda *et al*. [35] indicated *mcr*-4.3 in China from a human patient, but it did not show any colistin-resistance. Furthermore, Fernandes *et al*. [36] reported on a novel *mcr*-5.3 variant in South Africa from a horse that had not been previously treated by colistin. Hammerl *et al*. [37] also identified *mcr*-5.2 in Germany from swine fecal samples at farms and from cecal contents of swine at slaughter. Moreover, Wang *et al*. [38] investigated the presence of *mcr*-3.10 in a fecal sample from a duck in China. One study in Italy (2018) described the special diversity of six different *mcr* variants, with a high predominance of both *mcr*-1.1 and *mcr*-1.2 on conjugative IncX4 plasmids in *Salmonella* and in *E. coli* isolates from food-producing animals [18]. At the same time, *mcr*-1.13 was detected as a new variant within the chromosomes of *E. coli* from turkey and swine isolates. In addition, this study described *mcr*-3.2 and *mcr*-4.3 from cattle and *mcr*-4.2 from swine, where all of these variants were detected in *E. coli* [18]. Novel *mcr*-4.4 and *mcr*-4.5 variants were described in Spain, from pigs that were hosting five different isolates [19]. Finally, *mcr*-8 and *mcr*-8.4 were just identified by Wang *et al*. [39], in *Raoultella ornithinolytica* isolates, which are a member of the family from poultry samples in China.

## *Mcr*-gene Dissemination

A few years ago, colistin resistance in Enterobacteriaceae was considered uncommon, but that fact was changed when Liu *et al*. reported on a novel plasmid colistin resistance gene, *mcr*-1, from animal-originated *E. coli*, and they then found multi-resistance plasmids from animals, humans, and retail meat in *K. pneumoniae* and *E. coli* [7]. Nowadays, this resistant gene, *mcr*-gene, is disseminated, all over the world, and it threatens the therapeutical effectiveness against multidrug-resistant bacteria. Worryingly, these kinds of pathogens are untreatable with regular antibiotics [40]. Moreover, by tracking the *mcr* gene discoveries in the world, one can notice that the first *mcr* (*mcr*-1) was identified in Asia in 2016 [7], followed by many studies identifying the existence of the *mcr* gene in different places, and reported on a worldwide dissemination, namely, in Asia, Europe, North America, Africa, and the Middle East [29-31,41]. In addition, since 2015 up until the present, a huge number of researches has been conducted into the emergence and dissemination of the *mcr* gene, and this has resulted in nine different *mcr* variants and many subvariants [22,42,43]. Here, in this study, examples of the dissemination of the *mcr* gene have been demonstrated in different continents and with different bacterial species. In fact, *mcr*-1 was first reported in China by Liu *et al.*, but since then, this gene has been detected in several countries and from different sources, including natural and human-associated environments [44,45], food [46-48], animals [49,50], and humans [30,51], as shown in Table-2, and as modified from the Figure as published by Sun *et al*. [29], illustrating the pan *mcr*-1 distribution. In addition, *mcr*-1 was harboring in various species of the Enterobacteriaceae family, as has been indicated by many Epidemiological studies. Table-3 summarizes these bacterial species. Recently, several studies have shown how big the problem is in the Middle East. In August 2018, a study in Lebanon detected the existence of *mcr*-1 harbored by *E. coli* from swine fecal samples [52]. Another study in Lebanon by Hmede and Kassem investigated fresh fecal samples of broiler chickens and detected *mcr*-1 in *E. coli* [41]. Moreover, Eltai *et al*. [53] studied broiler chicken fecal samples in Qatar and they proved the presence of *mcr*-1 in *E. coli* isolates. Furthermore, the existence of the *mcr*-1 gene in colistin resistance Enterobacteriaceae in the Arabian Peninsula countries was reported by Sonnevend *et al*. [54] in four *E. coli* isolates of human origin; two isolates from Bahrain, one isolate from the Saudi Arabia and one isolate from the United Arab Emirates). In addition, *mcr*-3, as previously mentioned, was reported in China in 2017 from *E. coli* isolates [24]. In the same year, Litrup *et al*. [55] also identified *mcr*-3 positive isolates in Denmark from humans, harbored by *Salmonella* [55]. Nevertheless, Belaynehe *et al*. [56] reported on *mcr*-3 in *E. coli* from healthy animals in South Korea. Moreover, *mcr*-5 was first reported in 2017 in Germany from poultry and animal-derived food products [57], one year later, it was proved to presence in China pigs, and showed a horizontal transmission of the resistant gene among *Aeromonas hydrophila*, while it existed widely among the *Enterobacteriaceae*, *Pseudomonas*, and *Aeromonas* species. The threat of *mcr* gene dissemination comes from its horizontal transmission. Initially, it was believed that the *mcr* genes were transmitted from domestic animals by their milk, meat, and eggs to humans, or by direct contact [14,58]. Conversely, Villa *et al*. [59] proved that *mcr*-1 could be transmitted to *E. coli* strains and colonized in different hosts, such as in humans and pets, all within the same place. However, García *et al*. [19] showed that food-producing animals might have the ability to spread a cocktail of resistant *mcr*-genes, demonstrating an annoying threat to human health. On the other hand, the pan dissemination of *mcr*-1 has been connected with an elevation of human travel, as an explanation for the existence of the gene in enteric bacteria from European travelers returning to their home, before having visited countries with a high prevalence of *mcr*-1 in Asia, South America, and Africa [13,58]. Aquaculture is one of the most important food production practices worldwide. Some studies have focused on the aquaculture contribution to colistin resistance bacteria and dissemination. For example, Almeida *et al*. [60] reported on the isolation of *Enterobacter cloacae* from fish and shrimps having resistance to colistin, dependent on the minimal inhibitory concentration (MIC) results. Colistin resistance was reported in *Salmonella abony* with novel mutations on the chromosomal pmrA and pmrB genes by Antunes *et al*. [61]. Recently, Lv *et al*. [62] were the first to investigate that *mcr*-1 that was hosted in *E. coli* in China from a freshwater fish origin. They also thought that the existence of *mcr*-1 in retailed freshwater fish was a critical issue to consider, due to the possibility of gene dissemination globally by the aquatic international trade and that it would threaten human health through the food chain [62]. Partridge *et al*. [63] revealed that *mcr*-3 and *mcr*-7.1 from the *Aeromonas* species, *mcr*-4 from the *Shewanella* species, and *mcr*-5 from the *Legionella* species were all considered as aquatic bacteria. In addition, a study revealed that the geographic zones play a critical role in the dissemination of the *mcr* genes [64]. For example, the areas with limited aquaculture activities would have a significantly lower difference of *mcr*-1 carriage in human isolates than those areas with greater aquaculture industries [64].

**Table-2:** Global distribution of MCR-1 worldwide [29].

Continent	Country	Animal	Human	Environment
North America	USA	•	•	
	Canada		•	
South America	Venezuela	•		
	Colombia		•	
	Ecuador		•	
	Brazil	•	•	
	Bolivia			•
	Argentina		•	
Asia	China	•	•	•
	India		•	
	Pakistan	•	•	
	Oman		•	
	Saudi Arabia		•	
	Japan	•	•	
	Korea	•	•	
	Singapore		•	
	Malaysia	•	•	
	Thailand		•	
	Vietnam	•	•	
	LAOS	•	•	
Africa	Egypt	•	•	
	Tunisia	•		
	Algeria	•	•	
	South Africa	•	•	
Europe	Norway		•	
	Sedan		•	
	Estonia	•		
	Lithuania	•		
	Denmark		•	
	Germany	•	•	•
	Spain	•	•	•
	UK	•	•	
	France	•	•	
	Italy	•	•	
	Netherland	•	•	
	Portugal	•	•	
	Switzerland		•	•
	Bulgaria	•		
	Belgium		•	
Australia			•	

## Bacterial Resistance to Colistin

There are Enterobacteria isolates in humans, animals, and in the environment and this has been investigated and related to plasmid-mediated colistin that was encoded by the *mcr*-1 gene [65]. Table-3 represents the bacterial species and the specific types of *mcr* genes that are hosted [7,14,21,22,25-28,37,38,46,57,66-76]. Up-to-date, the presence of the *mcr*-1 gene has been proved in several bacterial species, for example, in *E. coli*, *K. pneumoniae*, *S. enterica*, *Enterobacter aerogenes*, *E. cloacae*, and more [77]. Besides, various types of bacteria have been identified to the harbor in more than one *mcr* gene; for example, *Salmonella* has been reported to host *mcr*-1, *mcr*-4, *mcr*-5, and *mcr*-9 [23,25,26,66]. Moreover, *E. coli* has been recognized as the superior host bacteria for the *mcr*-gene, by harboring *mcr*-1 up to *mcr*-5, with the chance for coexistence of more than one *mcr* gene [22,24-26,37]. Other studies have identified *K. pneumoniae* as a host for the new genes, such as *mcr*-7 and *mcr*-8, in addition to *mcr*-1 [7,21,22,67]. Up-to-date, *Moraxella pluranimalium*, which was investigated by AbuOun *et al*. [27], was the only bacteria that hosted the *mcr*-6 gene from animal isolates in Europe.

**Table-3:** Bacterial species and the relative *mcr* genes.

Bacteria	MCR gene									Country	Origin	References

	1	2	3	4	5	6	7	8	9			
*E. coli*	•	•	•	•	•					Asia, Europe, Africa	Animal, Human	[7,14,25,28,37,38,67,68]
*K. pneumonia*	•						•	•		Asia	Animal, Human	[7,21,^22^,^67^]
*Salmonella* spp.	•			•	•				•	Asia, Europe, USA	Animal, Food, Human	[23,25,26,66]
*M. pluranimalium*						•				Europe	Animal	[27]
*E. aerogenes*	•									Asia	Human	[69]
*E. cloacae*	•									Asia	Human	[69]
*C. sakazakii*	•									Asia	Animal	[70]
*S. sonnei*	•									Asia	Human	[71]
*Kluyvera* spp.	•									Asia	Sewage	[72]
*Citrobacter* spp.	•									Asia, South America	Food	[73,74]
*R. ornithinolytica*	•									Asia	Food	[46]
*A. hydrophila*					•					Asia	Animal	[57]
*A. caviae*			•							Asia	Animal	[38]
*P. mirabilis*			•							Asia	Animal	[38]
*A. baumannii*				•						Asia	Animal	[75]
*V. parahaemolyticus*	•									Asia	Food	[76]

*K. pneumoniae=Klebsiella pneumoniae*, *E. coli=Escherichia coli, M. pluranimalium=Moraxella pluranimalium*, *E. aerogenes=Enterobacter aerogenes*, *E. cloacae=Enterobacter cloacae*, *C. sakazakii=Cronobacter sakazakii*, *S. sonnei=Shigella sonnei*, *R. ornithinolytica=Raoultella ornithinolytica*, *A. hydrophila=Aeromonas hydrophila*, *A. caviae=Aeromonas caviae*, *P. mirabilis=Proteus mirabilis*, *A. baumannii=Acinetobacter baumannii*, *V. parahaemolyticus=Vibrio parahaemolyticus*

## The International Response to *mcr Gene* Dissemination

The emergence of the plasmid-mediated *mcr* gene has initiated the world’s awareness of the way of using colistin to treat the diseases caused by Enterobacteriaceae and, principally, to deal with the increasing applications of colistin, as a growth promoter in the veterinary field. The reduction in the overall use of colistin was the main idea for controlling the dissemination of the *mcr* genes internationally, as suggested by many researchers [78,79]. Recently, the World Health Organization added polymyxins as one of the critically important antimicrobial agents to be used in food-producing animals internationally [80]. In response to this, and to the predominant increase of common MDR bacterial infections in South Africa, they worked on the development of the “One Health-Based National Strategic Framework” for antibiotic resistance [81]. They also worked on the accomplishment plan [82], setting a program for the governance of stewardship for antibiotic use at different levels [83]. Moreover, The European Food Safety Authority (EFSA) applied the “One Health Approach,” due to the relationship of transferring bacteria from different sectors - humans, veterinary, and the environment. The EFSA also worked to increase their communication with food consumers because they can be part of the solution by changing attitudes and behaviors [84]. In June 2016, the European Medicines Agency developed a new concept on using colistin in European veterinary fields and they suggested that colistin should be carried out by the Antimicrobial Advice Ad Hoc Expert Group, which meant that colistin should be conserved for infections, where no other effective alternative drugs were available [85]. The American response took place in the US Centers for Disease Control and Prevention, by stating “the One Health concept recognizes that the health of humans is connected to the health of the animals and the environment” [84]. Robinson *et al*. [86], 2016 thought that the One Health Program depended on three main areas: Human health, animal fields, and environmental status. In 2016, the High Council for Public Health (HCSP) in France recommended the use of contact precautions with carriers of the *mcr*-1/*mcr*-2 Enterobacteriaceae strains [87]. The recommendations of the HCSP held four critical points. First, plasmid-mediated colistin resistance should be investigated in all carbapenemase-producing Enterobacteriaceae (CPE) strains. Second, by applying the hygiene precautions into two types; either by applying a specific action to observe the emerging highly resistant bacteria besides those carrying the *mcr*-1 gene [88] or by applying the 2009 SF2H guidelines, namely, “cross-transmission: Contact precautions” [89]. Third, the proper action when plasmid-mediated colistin is detected is to report the resistant gene into the nosocomial infection reporting system; then, all strains carrying the *mcr*-1*/mcr*-2 gene, and not only for the CPEs should be sent to the National Reference Center for Antibiotic Resistance. Finally, the French epidemiological situation can be improved by an assessment of the prevalence of colistin resistance and the presence of the *mcr*-1 resistance gene among the Enterobacteriaceae strains originating from the community and from hospital laboratory data. The epidemiological surveys might also be regulated by a national working group made up of experts from HCSP, ONERBA, the National Reference Center for Antibiotic Resistance in Clermont-Ferrand, and the government agency of Santé publique France. All of the previous recommendations that were aimed to manage the dissemination threat of plasmid-mediated colistin resistance within the Enterobacteriaceae strains should be kept by updating any reports of emergency of any *mcr* genes in infectious cases [87]. Furthermore, the researchers in the literature spotted a light on re-evaluating the dosage regimens of colistin for the treatment of lung infections. Therefore, Lin *et al*. [90] aimed to develop a mechanism-based PK/PD model (MBM) that would determine the time course of the colistin concentrations in the epithelial lining fluid, the plasma, and the bacterial concentrations, post the administration of different dosage regimens of colistin in neutropenic infected mice. Furthermore, Lin *et al*. [90] compared the newly developed MBM and a previously developed population PK model of aerosolized CMS and they formed colistin in critically ill patients, to predict the efficacy of inhalational dosage regimens of colistin (as CMS) in humans. Researchers from Italy strongly recommended, not only to reduce the overall usage of colistin but also to decrease the use of other different types of antimicrobial agents at a primary production level. This was to reduce the effects of complex mechanisms behind the multidrug resistance and the coselection of critically highly important antimicrobials while staying in a “Consumer Protection” and a “One Health” perspective [18]. Interestingly, Thakur and Gray believed that researchers will never recognize the spread of the AMR challenge and will only tackle AMR effectively if they harmonize the surveillance between nations [91]. They also suggested that surveillance should be an international “One Health” effort to solve this critical threat to humans, animals, and environmental health because the world has already reached the top point or even passed the top era of this problem [91].

## The Alternatives for Colistin

One of the most critical challenges to deal with infectious diseases nowadays is the limited treatment options due to two main points: First, the lack of development of new antimicrobial agents, and second, the persistent increase in global AMR [48,92]. Antibiotic resistance is believed to be a serious and growing global threat; specifically, the resistance to colistin is considered to be a great concern to the world community, due to the value of colistin, as it is the last choice available to treat multi-resistant Gram-negative bacteria [93]. By reducing the excessive use of antimicrobials, means to implement alternative measures, to limit the emergence and the spread of bacterial infections. Moreover, by increasing the awareness of people to stop the misuse of antibiotics and their overuse is critical. In this section, the strategies that are used to minimize antibiotic resistance will be reviewed, especially the antibiotic alternative options for reducing colistin resistance. One strategy has been to develop a better tolerated and more effective combination with the superior antimicrobial features of polymyxin, in addition to finding proper antibiotic combinations with colistin against polymyxin-non-susceptible Gram-negative bacterial infections. Vaara [94] reviewed four different programs that proved novel derivatives have better efficiency than the old polymyxins when applied to animal infection models; they were identified as Monash Cantab compound CA824, MicuRx compound 12, and compound NAB739 from northern antibiotics. These programs included three different programs that were superior to the known polymyxins in the rodent lung infection model with *Pseudomonas aeruginosa* and/or *Acinetobacter* *baumannii*. Interestingly, one of the programs showed a superior effect than did polymyxin B in mice infected by *A. baumannii*. The fourth program included compounds that were nearly ten-fold more effective in *E. coli* murine pyelonephritis than polymyxin B, which was compound NAB739 from northern antibiotics [94]. Moreover, to overcome the antibiotic resistance, a study by MacNair *et al*. [95] aimed to work in a new method using a combination of different antibiotics [95]. Briefly, they screened many Enterobacteriaceae that expressed *mcr*-1, against several antibiotics to decrease MIC in the presence of colistin. As a result, the greatest decline in MIC was reported by a combination of colistin and effective antibiotics against Gram-positive bacteria, such as rifampin and macrolides. These combinations were a successful treatment in two mouse models, against an *mcr*-1-positive *K. pneumoniae* infection [95]. These results are in agreement with those results as reported by Hu *et al*. [96], who proved the success of colistin combinations with azidothymidine, so as to treat antibiotic-resistant Enterobacteriaceae infections. On the other hand, there are many options available for reducing the use of colistin in animals by the use of non-antibiotic alternative products. There is a mounting interest in developing bacteriophage-based products for an administration to food animals as a new class of antimicrobial agents. Several studies have demonstrated that bacteriophages are “phages that are viruses capable of infecting bacteria,” in line with the ideas of antibiotic alternatives. For instance, Jeon *et al*. [97] recognized a novel *A. baumannii* lytic phage, the YMC 13/03/R2096 ABA BP (phage Βϕ-R2096), and the results strongly recommended that phage Βϕ-R2096 could be an alternative antibiotic agent to treat carbapenem-resistant *A. baumannii* infections [97]. Similar patterns of results were obtained by Prasanth Manohar *et al.*, when they studied the isolation and the characterization of the bacteriophages that effected *E. coli*, *K. pneumoniae*, and the *Enterobacter* species. The bacteriophages were named as *Escherichia virus myPSH2311, Klebsiella virus myPSH1235*, and *Enterobacter virus myPSH1140*. These three phage cocktails were effective against mixed bacterial populations that were resistant to meropenem and colistin [98]. Others have shown that some feed additives, like guanidine acetic acid, could be used as antibiotic alternatives, and they would significantly affect carcass characteristics and the economic traits of broiler chickens [99]. Some authors have also suggested the use of Artilysin^®^s, which are newly engineered enzyme-based experimental therapeutics that are effective against Gram-negative and Gram-positive pathogens. They could be used as bactericidal agents against all *E. coli* isolates that harbor the *mcr*-1 gene [100]. In addition, Art-175 could be a solution against bacteria and that may develop a pan drug resistance, due to its rapid and specific mode of action, by also decreasing the probability of inducing genetic resistance [101]. Recently, the main aim for a study by Otto *et al*. [102] was the non-antibiotic molecules in combination with polymyxin B. This study demonstrated a potential efficacy of three antidepressants (amitriptyline, imipramine, and sertraline) and four antipsychotics (chlorpromazine, clonazepam, haloperidol, and levomepromazine), together with polymyxin B, against 20 tested Gram-negative bacteria that displayed various resistance mechanisms, including the carbapenemases. From these results, it was clear that only sertraline, chlorpromazine, and levomepromazine had a synergistic effect with polymyxin B against the *A. baumannii, E. coli*, and *K. pneumoniae* isolates. Among all of the non-antibiotics, only spironolactone, which had a good efficacy against the *E. coli* isolates, showed non-toxic levels of a minimum concentration for synergy with polymyxin B [102]. Furthermore, Cheng *et al*. identified three pairs of two-drug combinations that showed synergistic effects with two known antibiotics against the *A. baumannii* strain AB5075, including azithromycin/5-fluorouracil, CS/fluspirilene, and CS/Bay 11-7082 [103]. In this section, the study has focused on the existing alternatives to colistin use in animals. However, in the past two decades, many types of research have been focused on the development of alternatives to antibiotics in agricultural animals, such as probiotics, prebiotics, enzymes, peptides, phytochemicals, and heavy metals, such as copper and zinc. Vaccines, bacteriophages, and many other alternatives in Table-4 summarize the most important types [104-114], the possible times of application when used in the most important animals. Many of these alternatives have already been applied as an alternative to colistin, and the other options still need to be studied for the possibility of applying them as alternatives to colistin, or other antibiotics in the field.

**Table-4:** Alternative products to colistin modified from the PEW Report [104].

Product type	Definition	Purpose of use			Animal species	References

		1	2	3		
In-feed enzyme	“Enzymes are biologically active proteins that break specific chemical bonds to release nutrients for further digestion and absorption”	•	•		Swine Chicken Turkey	[105]
Probiotics	A definition approved by FAO/WHO (2001) states that “Probiotics are mono or mixed cultures of live organisms which, when administered in adequate amounts confer a health benefit to the host.”	•	•	•	Cattle Swine Chicken Turkey	[106]
Prebiotics	A definition approved by (FAO, 2007) describes prebiotics as “non-viable feed components that confer a health benefit on the host associated with modulation of the microbiota”	•	•		Calves Swine Chicken Turkey	[107]
Antimicrobial peptides	“AMPs are small molecular weight proteins with broad-spectrum antimicrobial activity against bacteria, viruses, and fungi”	•	•	•	Cattle Swine Chicken	[108]
Organic acids	“Organic acids activity are short-chain acids (C1-C7) and are either simple monocarboxylic acids such as formic, acetic, propionic and butyric acids, or are carboxylic acids bearing a hydroxyl group (usually on the α carbon) such as lactic, malic, tartaric, and citric acids”	•	•		Cattle Swine Chicken Turkey	[109]
Phytochemicals (feed additives)	“Phytogenic are commonly defined as plant-derived compounds incorporated into diets to improve the productivity of livestock through amelioration of feed properties, promotion of the animals’ production performance, and improving the quality of food derived from those animals”	•	•	•	Cattle Swine Chicken Turkey	[110]
Heavy metals (copper, zinc)	“Heavy metals such as zinc and copper are naturally occurring and necessary as trace minerals in the diet but are extensively used in higher concentrations for growth promotion, and occasionally as therapy for enteric disease”	•		•	Cattle Swine Chicken Turkey	[111]
Vaccines	“Vaccines are substances used to mimic the development of naturally acquired immunity by inoculation of nonpathogenic but still immunogenic components of the pathogen in question, or closely related organisms”		•		Cattle Swine Chicken Turkey	[112]
Immune modulators	“The transfer of antibodies to elicit passive immune responses, are promising alternatives for disease prevention and potentially for treatment as well”		•	•	Cattle Swine Chicken Turkey	[104]
Bacteriophages	“Bacteriophages are viruses that infect and multiply in bacteria”		•	•	Cattle Swine Chicken Turkey	[113]
Predatory bacteria	“Predatory bacteria such as the “MDR Gram-negative bacteria have emerged as a serious threat to human and animal health. *Bdellovibrio* spp. and *Micavibrio* spp. are Gram-negative bacteria that prey on other Gram-negative bacteria”			•	In Experiment State	[114]
Cas9	“Cas9 and similar products work by reprogramming parts of the bacterial immune system (i.e., Cas9, a nuclease in the type II CRISPR system of bacteria) to selectively target specific parts of the bacterial genome (i.e., virulence factors), thereby selectively inactivating harmful bacteria that possess these virulence genes”			•	In Experiment State	[104]

1=Growth promotion, 2=Disease prevention, 3=Disease treatment. AMPs=Antimicrobial peptides, MDR=Multidrug-resistant

## Conclusion

Colistin resistance is a critical issue to deal with nowadays. Many studies have proved this resistance in several bacterial species and in different countries around the world. The *mcr* gene was identified as the responsible gene for unique colistin resistance because it is able to transmit horizontally from one bacterium to another and between animals, humans, and the environment. Most of the resistant bacteria were also featured as being MDR. In addition, the *mcr* variant genes were reported by many studies, and some of them showed resistance to colistin, while the others were susceptible. However, the scientific society has taken a response to reduce the negative effects of this resistance by applying some rules, such as forbidding the use of colistin, except for exceptional cases, and in the applied “One Health Approach.” Moreover, some researchers have launched a novel alternative to colistin, by the development of a new antibiotic, with better effects and with more tolerance than colistin, using antibiotic combinations with different antibiotics, or even with non-antibiotic particles. Overall, the antibiotic use of colistin must be reduced by establishing limits for its use. The current researchers hope that this review will help other researchers in building a better understanding of the colistin profile in different parts of the globe, such as in the emergence of its resistance and the proper actions to deal with this resistant problem. In addition, this study encourages them to work on papers about different detection methods for the colistin-resistant gene and titling the *mcr* genes in a well-set system.

## Authors’ Contributions

MHG: Gave the idea and prepared the outlines. MHG and SQS: Designed the tables. MHG and SQS: Contributed equally to the drafting of the manuscript. MHG: Reviewed the final draft. Both authors have read and approved the final version.
